# Computer-Aided Diagnosis for Early-Stage Lung Cancer Based on Longitudinal and Balanced Data

**DOI:** 10.1371/journal.pone.0063559

**Published:** 2013-05-15

**Authors:** Tao Sun, Regina Zhang, Jingjing Wang, Xia Li, Xiuhua Guo

**Affiliations:** 1 School of Public Health, Capital Medical University, Beijing, China; 2 College of Arts and Sciences, Emory University, Atlanta, Georgia, United States of America; Thomas Jefferson University, United States of America

## Abstract

**Background:**

Lung cancer is one of the most common forms of cancer resulting in over a million deaths per year worldwide. Typically, the problem can be approached by developing more discriminative diagnosis methods. In this paper, computer-aided diagnosis was used to facilitate the prediction of characteristics of solitary pulmonary nodules in CT of lungs to diagnose early-stage lung cancer.

**Methods:**

The synthetic minority over-sampling technique (SMOTE) was used to account for raw data in order to balance the original training data set. Curvelet-transformation textural features, together with 3 patient demographic characteristics, and 9 morphological features were used to establish a support vector machine (SVM) prediction model. Longitudinal data as the test data set was used to evaluate the classification performance of predicting early-stage lung cancer.

**Results:**

Using the SMOTE as a pre-processing procedure, the original training data was balanced with a ratio of malignant to benign cases of 1∶1. Accuracy based on cross-evaluation for the original unbalanced data and balanced data was 80% and 97%, respectively. Based on Curvelet-transformation textural features and other features, the SVM prediction model had good classification performance for early-stage lung cancer, with an area under the curve of the SVMs of 0.949 (P<0.001). Textural feature (standard deviation) showed benign cases had a higher change in the follow-up period than malignant cases.

**Conclusions:**

With textural features extracted from a Curvelet transformation and other parameters, a sensitive support vector machine prediction model can increase the rate of diagnosis for early-stage lung cancer. This scheme can be used as an auxiliary tool to differentiate between benign and malignant early-stage lung cancers in CT images.

## Introduction

Lung cancer, one of the most common cancer-related deaths, accounts for 1.1 million deaths annually worldwide [1]. Although attention has been paid to early stage predictions and diagnoses, prognosis remains very poor, with five-year survival rates ranging from 54% for Stage I to 10% for Stage III [2]. This emphasizes the need for a reliable early-stage prediction process that can prolong patients’ lives. Digital Computed Tomography (CT) is currently widely used for lung cancer in clinical practices. However, in CT images, lung cancer usually appears as solitary pulmonary nodule (SPN), and share similarities with those of several benign diseases [3]. By definition, the solitary pulmonary nodule (SPN) is a single, spherical, well-circumscribed, radiographic opacity that measures < = 3 cm in diameter and is surrounded completely by the aerated lung. There is no associated atelectasis, hilar enlargement, or pleural effusion.

With the development of science and technology, computer-aided diagnosis (CAD) has become an auxiliary tool. To our knowledge, using automated computerized methods, such as image texture analysis, to predict lung cancer has been reported widely [4]–[9]. Way et al. [4] extracted morphological, surface and texture features from 256 lung nodules, and established a linear discriminant analysis. A neural network-based computer-aided diagnosis method of lung nodule diagnosis by combining morphometry and perfusion characteristics to predict characteristics of solitary pulmonary nodules was introduced by Yeh et al. [5]. In another study, McCarville et al. [6] collected 81 pulmonary nodules, bases on CT findings to differ benign and malignant nature of pulmonary nodules in pediatric patients whereas Wang et al. [7] used the gray level co-occurrence matrix and the multi-level model to predict characteristics of pulmonary nodules. Lee et al. [8] used a two-step approach for feature selection classifier ensemble construction to facilitate the prediction of characteristics of pulmonary nodules. Zhu et al. [9] presented a method to find and select texture features of solitary pulmonary nodules (SPNs) detected by computed tomography (CT) and evaluate the performance of support vector machine (SVM)-based classifiers in differentiating benign from malignant SPNs. However, of these methods, none of them have aimed to predict early-stage lung cancer using texture analysis, in spite of the fact that it is crucial to prolong the lives of lung cancer patients by promptly resecting the cancer in its early-stage.

In previous study, they just used several morphological features (such as Mayo Clinic model and VA model) or textural features to predict the characteristic of nodules. In this paper, support vector machines (SVMs) were chosen as a prediction model, using a comprehensive set of textural features extracted by Curvelets [15] from CT images, patient demographic characteristics, and morphological features to predict early-stage lung cancer which appears as SPNs. To our knowledge, this is the first time that texture analysis was used to predict early-stage lung cancer and it is a useful undertaking.

## Materials

The data adopted in this paper was obtained from a cohort study. The cohort study was set up in 2009 and implemented in 4 hospitals. The decision on patient inclusion and exclusion was based on the results of the final diagnoses. The information in the CT images was accessed by 8 radiologists; meanwhile, conflicts in the final interpretation of the CT images were resolved by consensus discussion. A total of 360 cases were obtained from this cohort study. 317 cases (317/360) had only a time CT scan, where the patient was only scanned once, and the final diagnosis of malignant and benign cases was determined by either an operation or biopsy. 33 cases (33/360) had at least two CT scans with a follow-up period of 1 month to 2 years (patients were followed up until final diagnoses were available), and the final diagnosis of malignant and benign cases was determined by either an operation or biopsy. 10 (10/360) cases were excluded because of the lack of any final diagnosis.

CT scans were obtained using a 64-slice helical CT scanner (GE/Light speed ultra System CT99, USA) with a tube voltage of 120 kV and a current of 200 mA. The reconstruction thickness and reconstruction intervals for routine scanning were 0.625 mm. Data was reconstructed with a 512×512 matrix. In order to remove some other tissues (such as muscle, vessel and bone), all of the SPNs in the CT images were segmented manually to obtain a region of interest (ROI), and the textural features were extracted ROI by ROI. The region growing [10] algorithm, a popular tool for image segmentation, was used to remove any background pixels.

Training data included 317 cases which had only a time CT scan. A total of 10,108 ROIs were acquired from 317 patients, with 3131 benign ROI from 106 patients (58 males, 48 females) and 6977 malignant ROIs from 211 patients (125 males, 86 females). The details are as follows (See Table 1). The training data was used to establish a SVMs prediction model.

**Table 1:** Training data set.

		Number of cases	ROIs
Benign cases			
	Tuberculosis	33	1150
	Inflammatory pseudotumor	27	808
	Hamartoma	30	812
	Pulmonary interstitial edema	1	189
	Sclerosing hemangioma	9	93
	Clear cell tumor	1	11
	Chondroma	5	68
Malignant cases	Glandular cancer	155	5571
	Squamous carcinoma	47	1125
	Adenosquamous carcinoma	7	244
	Malignant carcinoid tumor	2	37

A total of 33 cases took at least two CT scans and the data set did not include SPN images of the last CT scan of each case. The reason why test data excluded the last CT scan of each case is that radiologists would make clinical diagnoses based on the last CT scan whatever correct or wrong and that the remaining data of the SPNs CT images which were hard to diagnose by radiologists were used to test the performance of a prediction model for early-stage lung cancer. This data is summarized in Table 2.

**Table 2:** Classification performance of the prediction model from the test data.

NO.	Actual diagnosis	CT diagnosis	Pathological diagnosis	Correct	NO.	Actual diagnosis	CT diagnosis	Pathological diagnosis	Correct
1	Hamartoma	Potentially malignant	Benign	YES	17	Glandular cancer	Potentially malignant	Malignant	YES
2	Hamartoma	Potentially malignant	Benign	YES	18	Glandular cancer	Potentially malignant	Malignant	YES
3	Hamartoma	Potentially malignant	Benign	YES	19	Glandular cancer	Potentially malignant	Benign	NO
4	Hamartoma	Potentially malignant	Benign	YES	20	Glandular cancer	Potentially malignant	Malignant	YES
5	Tuberculosis	Potentially malignant	Benign	YES	21	Glandular cancer	Potentially malignant	Malignant	YES
6	Tuberculosis	Potentially malignant	Benign	YES	22	Squamous carcinoma	Potentially malignant	Malignant	YES
7	Hamartoma	Potentially malignant	Benign	YES	23	Glandular cancer	Potentially malignant	Malignant	YES
8	Tuberculosis	Potentially malignant	Benign	YES	24	Glandular cancer	Potentially malignant	Malignant	YES
9	Inflammatory pseudotumor	Potentially malignant	Malignant	NO	25	Glandular cancer	Potentially malignant	Malignant	YES
10	Tuberculosis	Potentially malignant	Benign	YES	26	Glandular cancer	Potentially malignant	Benign	NO
11	Inflammatory pseudotumor	Potentially malignant	Benign	YES	27	Adenosquamous carcinoma	Potentially malignant	Malignant	YES
12	Tuberculosis	Potentially malignant	Malignant	NO	28	Glandular cancer	Potentially malignant	Malignant	YES
13	Tuberculosis	Potentially malignant	Benign	YES	29	Glandular cancer	Potentially malignant	Malignant	YES
14	Hamartoma	Potentially malignant	Benign	YES	30	Squamous carcinoma	Potentially malignant	Malignant	YES
15	Hamartoma	Potentially malignant	Benign	YES	31	Glandular cancer	Potentially malignant	Malignant	YES
16	Tuberculosis	Potentially malignant	Benign	YES	32	Glandular cancer	Potentially malignant	Malignant	YES
					33	Glandular cancer	Potentially malignant	Malignant	YES

## Methods

A set of textural features extracted by Curvelets from CT ROIs, demographic parameter and morphological features were used as input data to establish a SVMs prediction model. As a fact that one patient have several ROIs, so the malignance rate was used as the variable to draw a ROC curve. The malignance rate was defined as:
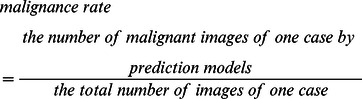
(1)


### Ethics Statement

This study was performed with ethics approval (Ethics Committee of Xuanwu Hospital, Capital Medical University, Approval Document NO. [2011] 01). Written consent was given by the patients.

### Synthetic Minority Over-sampling Technique (SMOTE)

The data acquired from the hospitals was unbalanced (the ratio of malignant to benign cases in the training data was 2∶1). Those data using for classification caused a bias on the training of classifiers and resulted in lower sensitivity during detection in the minority class examples [11]. If unbalanced data was used in this research study, the results would have high sensitivity and low specificity, which are undesirable results.

A data preprocessing method used to account for the unbalanced data consists of the following two categories [12]: under-sampling the majority class and over-sampling the minority class. Under-sampling methods are applied to remove some training majority class patterns to rebalance data sets, while over-sampling methods are used to form a new minority-class sample. Some researchers prefer over-sampling methods to under-sampling methods because using under-sampling methods risks the loss of majority class information.

The synthetic minority over-sampling technique (SMOTE) [13] is one such over-sampling method. Its main idea is to form new minority-class samples by interpolating between several minority-class examples that lie together. In the SMOTE, instead of mere data oriented duplicating, the positive class is over-sampled by creating synthetic instances in the feature space formed by the positive instances. For every minority example, its k (which is set to 5 in SMOTE) nearest neighbors of the same class are calculated, then some examples are randomly selected from them according to the over-sampling rate. After that, new synthetic examples are generated along the line between the minority example and selected nearest neighbors.

### Texture Extraction

Texture is a fundamental characteristic of the digital images as it usually reflects the structure of the pictured objects. Image feature extraction is an important step in image processing techniques.

The Wavelet transformation, a textural features extraction method, provides a multi-resolution and non-redundant representation of signals with an exact reconstruction capability, and forms a precise and uniform framework for the space–frequency analysis. Although Wavelets perform very well for objects with point singularities, they are not adequate for representing 1D singularity [14]–[15]. In 2000, Candes and Donoho [16] developed the Curvelet, a type of second generation Wavelets. As an extension of the Wavelet multiscale analysis framework, Curvelets can effectively deal with linear singularities in 2D signals [14]. The Curvelet transformation is defined as an effective tool for finding curves at multiple resolution levels. Several studies using Curvelet transformations in image processing have shown that Curvelet transformations yield better results [17]–[19].

Based on the Curvelet transformation, fourteen CT image textural features of pulmonary nodules were extracted: Entropy, Mean, Correlation, Energy, Homogeneity, Standard Deviation, Maximum Probability, Inverse Difference Moment, Cluster Tendency, Inertia, Sum-Mean, Difference-Mean, Sum-Entropy, and Difference-Entropy. As a pre-process for classification, a Curvelet transformation produced a representation of the pulmonary nodules of CT images through multi-scale level decomposition. The three scales’ Curvelet coefficients matrices (the coarse layer, the detail layer, and the fine layer) were chosen as candidates. ROI images were decomposed into 34 sub-bands, resulting in the extraction of 476 textural features from each ROI.

### Survey of Clinical Parameters

Three demographic parameters (age, gender, and smoking habits) were obtained from medical histories. 9 morphological features (including substantial changes, density of the SPNs, the presence of spicules, caverns, vacuoles, lobulation, calcification and ground glass in the SPNs, and area) were reported by experienced radiologists according to the SPNs.

### Prediction Model

As suggested by a large body of literature to date, support vector machines can be considered good algorithms for classification in some research fields [20]–[22]. In a previous study, the same results were demonstrated by our group [23].

The support vector machine (SVM) is described as a popular classifier based on the structural risk minimization principle. Compared to other classifiers, the SVM aims to find the hyperplane that maximizes the distance from the hyperplane to the nearest examples in each class. Given a set of training vectors (l in total) belonging to separate classes 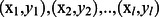, 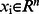 denotes the *i*th input vector and 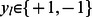 is the corresponding desired output. The maximal margin classifier seeks to find a hyperplane 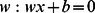 to separate the training data. In the possible hyperplanes, only one maximizes the margin (the distance between the hyperplane and the nearest data point of each class). The support vectors denote the points lying on the margin border. The solution to the classification is given by the decision function:
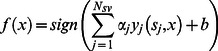
(2)


Where 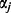 is the positive Lagrange multiplier, 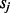 is the support vectors (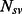 in total), and 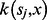 is the function for the convolution of the kernel of the decision function.

R 2.14.0 software was used to implement the support vector machines and the SMOTE. The radial basis function kernel was used as the kernel of the SVMs in this study.

## Results

### SMOTE for Pre-processing the Unbalanced Data Set

The distribution of 3 demographic parameters is shown in Table 3. The original training data included images of 3131 benign ROIs and 6977 malignant ROIs, with a ratio of malignant to benign cases of 2∶1. Using the SMOTE as a pre-processing procedure, new data including the textural texture, demographic parameters and morphological features was generated, and the final training data included observations of 9393 benign ROIs and 9393 malignant ROIs.

**Table 3:** The distribution of the three demographic parameters between benign and malignant cases.

		Benign	Malignance	Statistic	*P*
Smokinghabits	N (Missing)	106(0)	212(0)	2.79	0.0949
	No (%)	64(60.38)	107(50.47)		
	Yes (%)	42(39.62)	105(49.53)		
Age	N (Missing)	106(0)	212(0)	46.37	<0.0001
	Mean (Std)	50.8(13.26)	62(11.54)		
	Median (Q1,Q3)	50.5(42,60)	63(54,72)		
Sex	N (Missing)	106(0)	212(0)	0.78	0.3766
	Female (%)	48(45.28)	85(40.09)		
	Male (%)	58(54.72)	127(59.91)		

### Prediction Results

In order to test the SVM model based on balanced data whether it was sensitive to lung cancer, two methods were used: 10-fold cross-evaluation and new testing data evaluation.

Accuracy based on 10-fold cross-evaluation for the original unbalanced data and the balanced data was 80% and 97%, respectively. It was proven that the SMOTE algorithm would greatly increase the performance of the prediction model.

**Figure 1:**
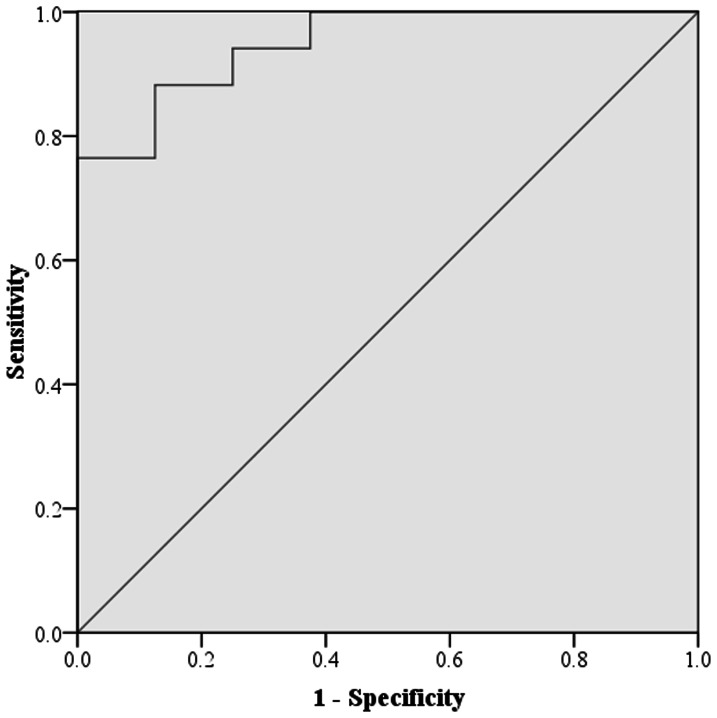
ROC curve created by SVMs.

33 cases (17 malignant cases, 16 benign cases) were chosen as test data to evaluate the classification performance for early-stage lung cancer. The SVM prediction model was successfully established using 488 textural features. The information about the cases was analyzed, and the malignance rate (Formula 1) was adopted as the independent variable to draw ROC curves, with the results presented in Figure 1. The area under the curve of the SVM was 0.949 (*P*<0.001, accuracy was 15/17 for malignant cases, 14/16 for benign cases). This result is summarized in Table 2. To test data in this study, every case had a CT diagnosis before operations and the results are shown in Table 2. CT diagnoses of 33 cases were all potentially malignant indicating that although through a period of follow-up time it is rather difficult to make a clear clinical decision.

**Figure 2:**
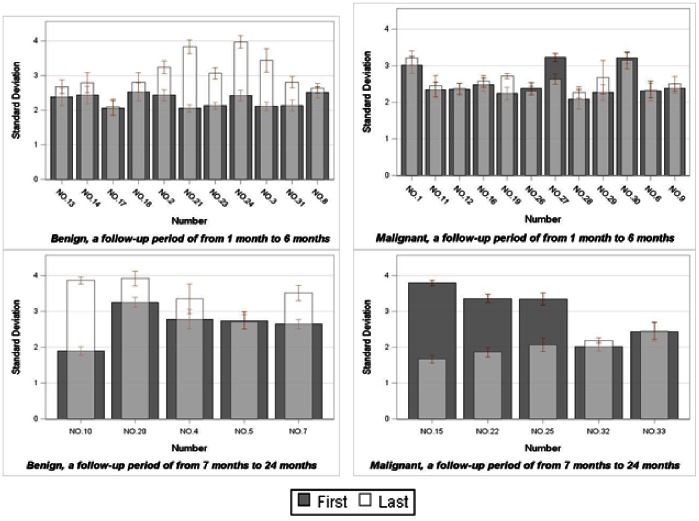
Change of textural features between the first CT scan and the last CT scan.

Also we did assess the change of textural features between the first CT scan and the last CT scan based on the test data set. We found the Curvelet textural feature (Standard Deviation) had a great difference between benign and malignant cases. Figure 2 demonstrates the change in trend of the textural feature (Standard Deviation) average value.

## Discussion

Currently, the incidence and mortality rates of lung cancer have ranked first among various tumors. The use of CT scans is common in clinical practices to distinguish between benign SPNs and malignant tumors. A meta-analysis [24] found that it has a pooled sensitivity of 0.57 (95% confidence interval, 0.49 to 0.66) and a pooled specificity of 0.82 (95% confidence interval, 0.77 to 0.86) for lung cancer using CT scans. All the above researches focused on lung cancer, and not on early-stage lung cancer. Thus, sensitivity and specificity for early-stage lung cancer could be poorer. Based on clinical practice, a high proportion of patients with suspicious benign conditions who could not exclude a possible malignancy would require further investigations or surgery, which would increase the burden on patients. Computer-aided diagnosis (CAD) technology has become more prevalent in assisting radiologists with making diagnoses. To our knowledge, researches on SPN image analysis discuss the prediction of the characteristics of lung cancer using texture analysis, not early-stage lung cancers which have more significant clinical value. In this study, longitudinal data was used as test data to evaluate the classification performance of the SVM prediction model for early-stage lung cancer. The area under the curve of the SVM was 0.949 (*P*<0.001), and the model has potential competence to predict early-stage lung cancer. Related literature has not yet been reported.

The data obtained from the hospitals was unbalanced. Using unbalanced data may cause a lower specificity when predicting benign cases. In this study, the SMOTE, an over-sampling method, was used as the pre-processing procedure to balance the data, and the classification performance (accuracy) of the prediction model had a great improvement from 80% to 97%. Thus, the SMOTE is a useful method to account for unbalanced data and can improve the capability of the models.

Several methods for extracting the textural features of images have been developed. One of the most popular methods is a Wavelet which is being widely used in the processing of medical images [14]–[15]. Compared to Wavelets, Curvelet transformations may provide stable, efficient, and near-optimal representations of smooth objects having discontinuities along smooth curves [14]. As a fundamental characteristic of the digital images, textural features usually reflect the microcosmic structure of the pictured objects, overlooking the macroscopic characteristics of the cases. In this paper, textural features extracted by Curvelets, in addition to 3 patient characteristics and 9 morphological features which were applied to describe macroscopic characteristics of tissues, were used as input variables to establish a SVMs prediction model. This scheme is sensitive to early-stage lung cancer and can therefore increase the accuracy rate of diagnosis.

In this study, we found the Curvelet textural feature, Standard Deviation, had a great difference between benign and malignant cases. Although all the cases did not have the same date for the previous CT scan, the textural feature (Standard Deviation) of benign cases had an obvious increase from the first CT scan to the last CT scan in most cases, but it was relatively steady in malignant cases. This result could be helpful as a clue to find a biomarker for lung cancer.

For 33 cases, the average CT scan per case was 3.2 times. The mean, median, interquartile range and standard deviation of follow-up time was 6.9, 2.0, 8.0 and 11.0 months, respectively. If the method involved in this paper can be used in clinical practice to help radiologists for decision making, the time for diagnoses will shorten by 6.9 months and save the cost of 2.2 CT scans (in Beijing China, the cost of 2.2 CT scans is about 1,000 RMB). Based on a meta-analysis [25], direct economic cost for lung cancer patients is different, ranging from 18,019.4 RMB per person for Stage I to 3,2534.0 RMB per person for Stage IV RMB per person in China and it is increasing year after year. Mental burden on patients and indirect economic cost are also important. China is one of the countries with the highest suicide rate among cancer patients in the world. Thus, if the scheme introduced in this study is used in clinical practice, it can reduce economic and mental burden on patients and prolong time of lung cancer patients. The architectures of the SVM and Curvelets are simple, redressed easily, and are appropriate for software design. It might be used in daily radiological practice because of its advantage in not far future.

There are, however, limitations involved in this study. The time interval between the first CT scan and the last CT scan is different across patients.
